# The association of religion with maternal and child health outcomes in South Asian countries

**DOI:** 10.1371/journal.pone.0271165

**Published:** 2022-07-12

**Authors:** Aparna G. Kachoria, Mohammad Yousuf Mubarak, Awnish K. Singh, Rachael Somers, Saleh Shah, Abram L. Wagner

**Affiliations:** 1 Department of Maternal and Child Health, Gillings School of Global Public Health, University of North Carolina, Chapel Hill, North Carolina, United States of America; 2 Microbiology Department, Kabul University of Medical Science, Kabul, Afghanistan; 3 National Technical Advisory Group on Immunization Secretariat, Ministry of Health and Family Welfare, National Institute of Health and Family Welfare, New Delhi, India; 4 Superior University, Lahore, Pakistan; PLOS: Public Library of Science, UNITED KINGDOM

## Abstract

**Objective:**

Theological beliefs play an important role in cultural norms and could impact women’s prenatal and postpartum decisions in South Asia, which has a high burden of disease in children and pregnant women. The aim of this study is to identify any associations religion may have in affecting a woman’s decision-making ability, and how that in turn affects maternal and child health, at a group level in multiple South Asian countries.

**Study design:**

Cross-sectional study utilizing secondary data analysis.

**Methods:**

We used Demographic and Health Surveys (DHS) between 2014 and 2018 in Afghanistan, Bangladesh, India, Maldives, Myanmar, Nepal, and Pakistan. Not every country’s survey asked about religion, so we imputed these results based on Census data. We assessed maternal and child health through a composite coverage index (CCI), which accounts for family planning, attendance of a skilled attendant at birth, antenatal care, BCG vaccinations, 3 doses of diphtheria-tetanus-pertussis vaccine, measles vaccine, oral rehydration therapy, and seeking care if the child has pneumonia. The relationship between religion, women’s empowerment, and CCI was assessed through linear regression models.

**Results:**

The sample included 57,972 mothers who had children aged 12–23 months. CCI is observed to be affected by family income, in addition to religion and country. CCI was higher in Hindus (2.8%, 95% CI: 2.4%, 3.1%) and Buddhists (2.0%, 95% CI: 1.2%, 2.9%) than Muslims. Mother’s age, education, income, decision-making autonomy, and attitude towards beatings were all related to CCI. In a model stratified by religion, age, education, and income were significant predictors of CCI for both Muslims and non-Muslims, but were more impactful among Muslims.

**Conclusion:**

Though multiple imputation had to be used to fill in gaps in religion data, this study demonstrates that maternal and child health outcomes continue to be a concern in South Asia, especially for Muslim women. Given the importance of religious beliefs, utilizing a simple indicator, such as the CCI could be helpful for monitoring these outcomes and provides a tangible first step for communities to address gaps in care resulting from disparities in maternal empowerment.

## Introduction

Maternal and child health outcomes are a core focus of the United Nations (UN) Sustainable Development Goal 3 [1]. With over 300,000 deaths around the world due to complications of pregnancy and childbirth, maternal health is an important indicator of community health and carries downstream effects for children’s health; the under-5 mortality rate, while decreasing globally, still remains at 39 deaths/1,000 live births [2–4].

A simplified index that incorporates different health outcomes can allow us to better understand the extent and geographical distribution of maternal and child health problems. One such measure is the Composite Coverage Index (CCI) [5]. CCI is a monitoring measure that takes the weighted average of eight different preventive interventions in the maternal and child health spectrum [5]. These measures include family planning, attendance of a skilled attendant at birth, antenatal care, BCG vaccinations, 3 doses of diphtheria-tetanus-pertussis vaccine, measles vaccine, oral rehydration therapy, and seeking care if the child has pneumonia [5]. These are all variables asked widely in the Demographic Health Survey (DHS) in multiple countries and provide an accessible window into the status of maternal and child health as relates to other factors that may influence outcomes in this field. Since CCI appeared as a tool, it has been used as an effective comparison tool for use between countries to better understand healthcare both broadly, and specifically to study certain outcomes such as maternal and child health at an aggregate level [6, 7].

South Asian countries continue to have relatively adverse maternal and child health outcomes. Studies have found high rates of risk factors during pregnancy and childhood, including underweight pregnant women and children with developmental delays [8, 9], low rates of vaccination coverage [10], and malnutrition [8]. Many of these problems, including childhood illnesses such as diarrhea and pneumonia, can be compounded by poor sanitation, making it even more difficult to adequately prevent and treat disease [11]. However, there is incredible regional diversity both between and within South Asian countries. For example, Bangladesh has relatively high vaccination coverage [12], as do certain states within India [13]. Similarly, in both India and Bangladesh, regional variation is common regarding how women seek treatment if their child develops an acute respiratory infection [14, 15]. Interventions to address maternal and child health in South Asia aim to address shortfalls across the region and target both antenatal and postpartum interventions for mother and child. Given that that the fertility rate is quite high in South Asia– 2.41 births per woman–and with a crude birth rate of 19.65 per 1,000 people in the region—addressing factors that influence decision making for both mother and child are necessary for promoting positive health outcomes [16].

Religion and culture often inform health decisions [17, 18]. South Asia is religiously diverse, and as a region, hosts the world’s largest populations of both Muslims and Hindus [19]. It is also the birthplace of Buddhism, Sikhism, and Jainism and contains large numbers of Christians and other religious groups. Religion could influence maternal and child health outcomes in different ways. For example, although Muslim religious councils as a whole have emphasized the importance of vaccination, there have been concerns raised by other councils that some vaccines, particularly the measles vaccine, may contain *haram* material [20]. Religion may also function as a space for women to explore their spirituality and feel empowered [21]. However, it is also possible that, if women are dependent on a male partner for seeking antenatal care and/or child health services, that religion could negatively impact maternal and/or child health [22].

As a populous, diverse region, South Asia offers the chance to better understand the role of religion in maternal and child health. The aim of this study is to identify how religion at a group level affects CCI, and whether this pathway can be explained by differential norms of maternal decision-making or wealth among religious groups within the country. As an outcome from this work, more targeted interventions that are culturally and religiously sensitive can be utilized to ensure that women and children are receiving appropriate care that improves their overall health. Multiple studies have aimed to quantify maternal and child health outcomes in simplified ways to bring about effective change, but more information is needed to understand the factors at play that drive decisions that women make in their care and in the care of their child(ren) [5–13, 17, 18]. With a better understanding of social and cultural norms that drive decision making, and with the ease of identifying health outcomes broadly with use of the CCI, it will be helpful in driving implementation efforts that aim to reduce disparities and poor maternal and child health outcomes in South Asia.

## Methods

### Study population

This cross-sectional study utilizes a secondary data analysis approach with data from the Demographic and Health Survey (DHS). The countries and years of survey data collection utilized in this study are described in Table 1.

**Table 1:** DHS datasets and sample size.

DHS Dataset	Country	Year	Original sample size	Final sample size
DHS VII	Afghanistan	2015/2016	15,698	5,406
	Bangladesh	2014	3,825	1,529
	India	2015/2016	124,525	46,517
	Maldives	2016/2017	1,522	571
	Myanmar	2015/2016	2,287	879
	Nepal	2016	2,386	974
	Pakistan	2017/2018	5,133	2,096

Note: Due to lack of recent data, Sri Lanka was excluded from this study.

### Composite coverage index

The main outcome variable of this study is the Composite Coverage Index (CCI), which was defined by Wehrmeister et al. in a WHO Bulletin in 2016 [5]. This measure is a weighted average, calculated between 0 and 1, of eight different preventative measures that are indicative of maternal and child health. The measures include indicators of reproductive care, maternal care, and children’s health including immunization status and management of illness. Specifically, the CCI is calculated based on percent coverage of: family planning coverage (FPC), skilled attendant present at birth (SBA), antenatal care (ANC), BCG (bacille Calmette-Guerin) vaccination for tuberculosis, 3 DPT (diphtheria, pertussis (whooping cough), and tetanus) vaccinations, measles vaccination (MSL), oral rehydration therapy for diarrhea (ORT), and seeking care for childhood pneumonia (CAREP). Wehrmeister et al. used this equation to calculate CCI:

$$CCI=\frac{1}{4}\left(FPC+\frac{SBA+ANC1}{2}+\frac{2(DPT3+BCG+MSL)}{4}+\frac{ORT+CAREP}{2}\right)$$

### Independent variables

Religion was the main independent variable in this study. Participants in most countries responded directly to this question when surveyed for the DHS. However, in Afghanistan, Maldives, Pakistan, and Myanmar, this question was not asked. To proceed with the analysis, a few assumptions were made based on census and available data. For Maldives, the government interpretation of the Constitution stipulates that all citizens follow Islam, and it is illegal to practice another religion [23]. Similarly, the Afghanistan constitution declares the country as an Islamic Republic [24]. As a result, for Afghanistan and Maldives, we imputed everyone to be Muslim. For Pakistan and Myanmar, we imputed an individuals’ religion based on the proportion who belonged to a given religious group by administrative unit based on the most recent Census [25, 26]. For example, if the proportion of the population who was Muslim in a given province was 90%, then we imputed their probability of being Muslim was 90% in a Bernoulli distribution. We created 100 imputations per individuals and split collapsed individuals into four different categories: Muslim, Hindu, Christian, or Buddhist and others.

We were also interested in maternal empowerment, which we define as a woman’s ability to make decisions about her reproductive health and the decision to have children. The variables chosen as measures of maternal empowerment in this analysis are knowledge of ovulatory cycle, the interval between marriage and the birth of the first child, whether the current pregnancy was wanted, who, between partners, is the decision maker for using contraception, current contraception method, age at first physical intimacy, and whether a woman felt her husband was justified in beating her for any combination of five reasons as per the DHS: wife goes out without telling husband, wife neglects children, wife argues with husband, wife refuses to have physical intimacy with husband, and/or when wife burns the food. Each variable was recategorized into dichotomous structure for determination of degree of empowerment. Variables were selected based on availability in the DHS datasets for each country and a priori literature about maternal empowerment in South Asia.

In our analyses, we also controlled for family income and maternal education. Family income was derived from the wealth index using a previous formula [27]. Briefly, within a given wealth index quintile for a country, everyone was imputed to have the same income. The average household income was defined as the gross national income / capita, adjusted to 2011 purchasing power parity multiplied by the average household size, multiplied by the national consumption share. We assumed natural log transformation of household income was distributed on a normal scale, and the standard deviation was calculated using the Gini coefficient as previously defined [28]. This measure of absolute income allows for better comparability of wealth statuses across countries.

### Statistical analysis

Analyses that incorporated religion used imputed values and appropriate statistical techniques using *PROC MI* in SAS version 9.4 (SAS Institute, Cary, NC, USA). We used DHS weights that had been re-weighted to account for different annual birth cohort sizes across the seven countries. To compare maternal and child health characteristics between Muslims and non-Muslims, we constructed separate logistic regression models for each outcome, with the model also adjusted for child’s sex, mother’s age, mother’s education, and family income. Subsequently, we regress the CCI on child’s sex, mother’s age, mother’s religion, mother’s education, decision-making autonomy, belief in justifiability of domestic violence, and the family’s income. These variables were chosen *a priori* to be in the model. Subsequently, we stratified the model such that there was an interaction term between Muslim religion and every other variable. We stratified the model to understand if decision-making varied between Muslims and non-Muslims. The p-value for the interaction terms in these models indicate whether the strength of association between the independent variable and CCI differed by religion. We treated p-values <0.05 as significant. To account for multiple testing in the separate logistic regression models of maternal and child health characteristics, we corrected the p-value using the Holm-Bonferroni method.

### Ethical approval

This study was deemed exempt by the University of Michigan Health Sciences and Behavioral Sciences Institutional Review Board because it uses secondary data (#HUM00162698). DHS data utilizes an ICF IRB review process both within the US and the host country.

## Results

**Fig 1:**
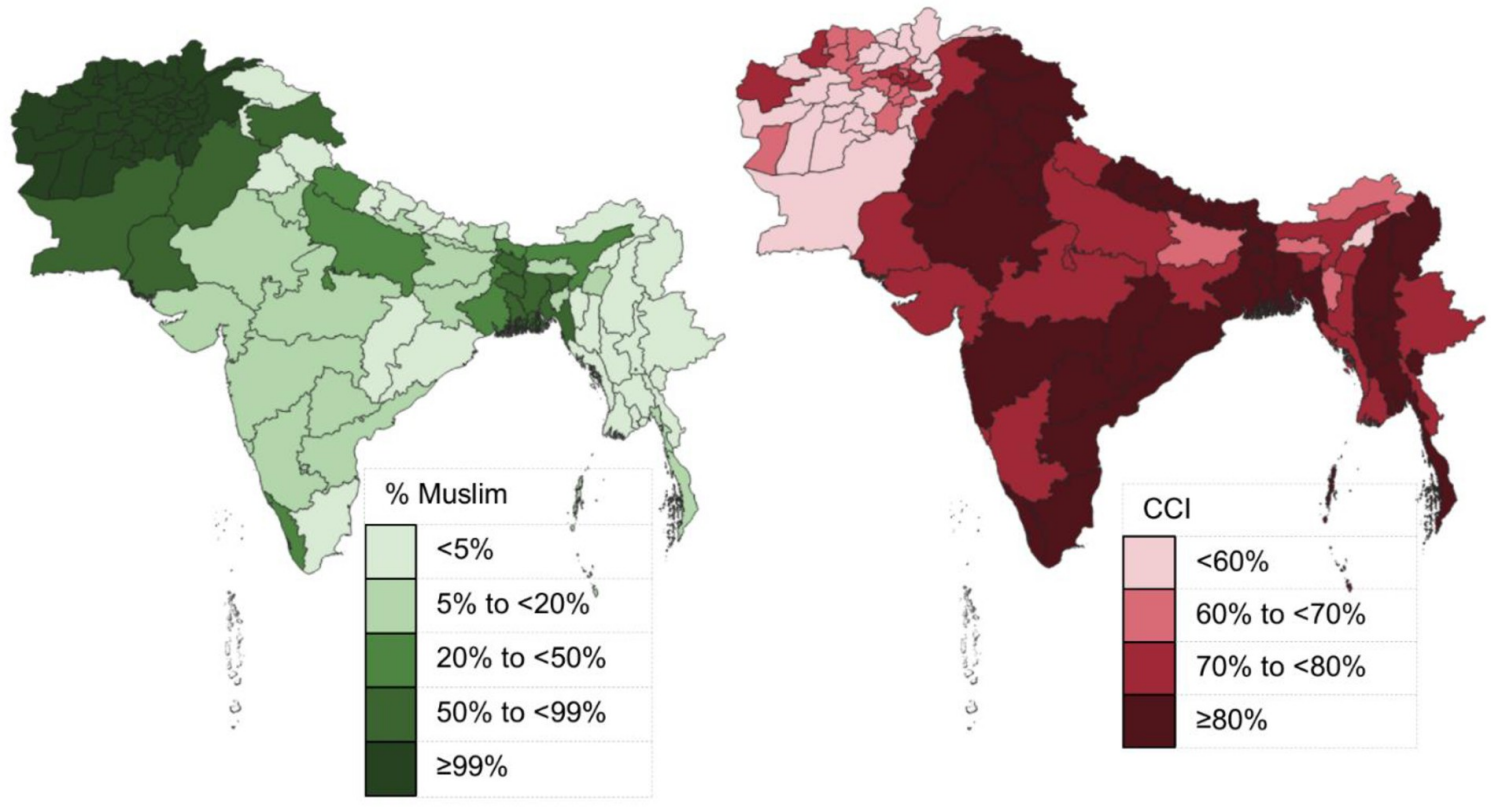
Choropleth maps of the proportion Muslim and the mean composite coverage index (CCI) across regions in 7 South Asian countries. Made with Natural Earth naturalearthdata.com.

The DHS data provided a large sample with multiple demographic characteristics to work with. These characteristics are summarized in Table 2. Overall, the sample included 57,972 mothers who had children aged 12–23 months, across all countries (Afghanistan, Bangladesh, India, Maldives, Myanmar, Nepal, and Pakistan). The distribution of Muslim respondents is illustrated in Fig 1. India and Nepal are majority Hindu nations, and Myanmar is a majority Buddhist nation, though each of these nations also had many mothers who observed other religions.

**Table 2:** Distribution of demographic and autonomy characteristics across 7 South Asian countries, among mothers of children 12–23 months old.

		All countries (%)	Afghanistan (%)	Bangladesh (%)	India (%)	Myanmar (%)	Maldives (%)	Nepal (%)	Pakistan (%)
	Total	n = 57,972	n = 5,406	n = 1,529	n = 46,517	n = 879	n = 571	n = 974	n = 2,096
**Child’s sex**									
** Female**	27,686	47.8%	49.2%	47.0%	47.8%	44.4%	48.1%	45.1%	48.8%
** Male**	30,286	52.2%	50.8%	53.0%	52.2%	55.6%	51.9%	54.9%	51.2%
**Mother’s age**									
** 15–19**	24,53	5.7%	5.7%	19.7%	3.9%	3.0%	0.4%	11.5%	3.3%
** 20–24**	20,217	34.8%	29.2%	35.6%	38.8%	21.3%	17.3%	37.9%	22.7%
** 25–29**	20,412	34.5%	31.2%	26.4%	36.5%	27.9%	30.2%	32.1%	33.5%
** 30–34**	9,698	16.9%	15.7%	13.5%	14.7%	25.1%	30.1%	13.7%	26.5%
** 35–39**	3,861	6.3%	11.7%	3.9%	4.9%	16.1%	17.7%	3.0%	11.3%
** 40–49**	1,331	1.8%	6.5%	0.9%	1.3%	6.7%	4.4%	1.9%	2.8%
**Mother’s religion**									
** Muslim**	16,948	41.2%	100.0%	92.4%	16.9%	2.2%	100.0%	7.0%	96.3%
** Hindu**	34,374	52.7%	0.0%	5.8%	78.3%	0.4%	0.0%	85.1%	1.7%
** Christian**	4,033	1.9%	0.0%	0.2%	2.1%	6.8%	0.0%	2.6%	1.7%
** Buddhist or other**	2,607	4.2%	0.0%	1.6%	2.7%	89.3%	0.0%	5.3%	0.6%
**Mother’s education**									
** None**	19,397	30.5%	80.8%	12.6%	27.4%	14.2%	1.0%	30.3%	45.8%
** Primary**	8,315	16.4%	8.1%	28.4%	13.9%	45.9%	14.7%	20.6%	15.3%
** Secondary**	24,405	41.5%	8.8%	49.4%	47.1%	31.6%	62.4%	34.7%	24.5%
** Tertiary**	5,855	11.7%	2.2%	9.6%	11.8%	8.3%	21.9%	14.4%	14.5%
**Decision maker for contraception use**									
** Yes**	20,595	40.7%	22.9%	65.2%	38.0%	61.3%	23.6%	39.0%	36.2%
** No**	37,377	59.3%	77.1%	34.8%	62.0%	38.7%	76.4%	61.0%	63.8%
**Believes beatings are justified**									
** Yes**	10,441	20.7%	85.8%	28.9%	8.0%	57.5%	18.9%	28.4%	44.1%
** No**	47,531	79.3%	14.2%	71.1%	92.1%	42.5%	81.1%	71.6%	55.9%
**Family income, USD (mean)**		25,172	18,657	14,468	23,628	17,793	73,996	11,920	40,637
**CCI (mean)**		78.4%	62.3%	82.8%	78.4%	81.0%	78.7%	89.0%	76.9%

Looking specifically at the maternal empowerment and CCI variables in Table 3, there are significant differences in outcomes between Muslim and non-Muslim mothers. These variables are adjusted for child’s sex, mother’s age, mother’s education, and family income. There were significant differences in maternal empowerment in that Muslim women were less likely to understand ovulation, have a smaller interval between marriage and the birth of the first child, and more likely to justify being beaten by their husbands (p < 0.0001). Additionally, observing CCI outcomes, non-Muslim mothers were more likely to have a skilled birth attendant present during the birth of the child, receive antenatal care, or obtain any of the vaccines–BCG, DPT, and measles–for their child (p < 0.0001). The geographic diversity in CCI is shown in Fig 1.

**Table 3:** Maternal and child health characteristics in Muslims and non-Muslims in 7 South Asian countries.

Characteristic	% in non-Muslims	% in Muslims	P-value*
**Knowledge of ovulation**	19.5%	14.4%	< .0001
**Marriage to first birth interval**	82.0%	74.4%	< .0001
**Current pregnancy wanted**	8.2%	8.7%	1
**Current use of contraception**	20.1%	33.2%	< .0001
**Woman’s age at first sex (>18)**	42.6%	45.5%	< .0001
**Decision maker for using contraception**	39.8%	42.0%	< .0001
**Does not believe beating is justified**	88.7%	66.0%	< .0001
**Family planning–use and intentions**	57.2%	66.2%	< .0001
**Skilled attendant present at birth**	83.8%	64.7%	< .0001
**Received at least 1 antenatal care visit**	84.2%	81.5%	< .0001
**Child received bCG vaccination**	93.0%	89.1%	< .0001
**Child received 3 DPT vaccinations**	80.0%	76.8%	0.0070
**Child received measles vaccination**	82.9%	74.6%	< .0001
**Child received ORT for diarrhea**	93.8%	89.9%	< .0001
**Child received medical care for pneumonia**	93.1%	85.4%	< .0001

*From religion parameter in multivariable models, also adjusted for child’s sex, mother’s age, mother’s education, and family income.

P-values adjusted for multiple testing with the Holm-Bonferroni method.

**Fig 2:**
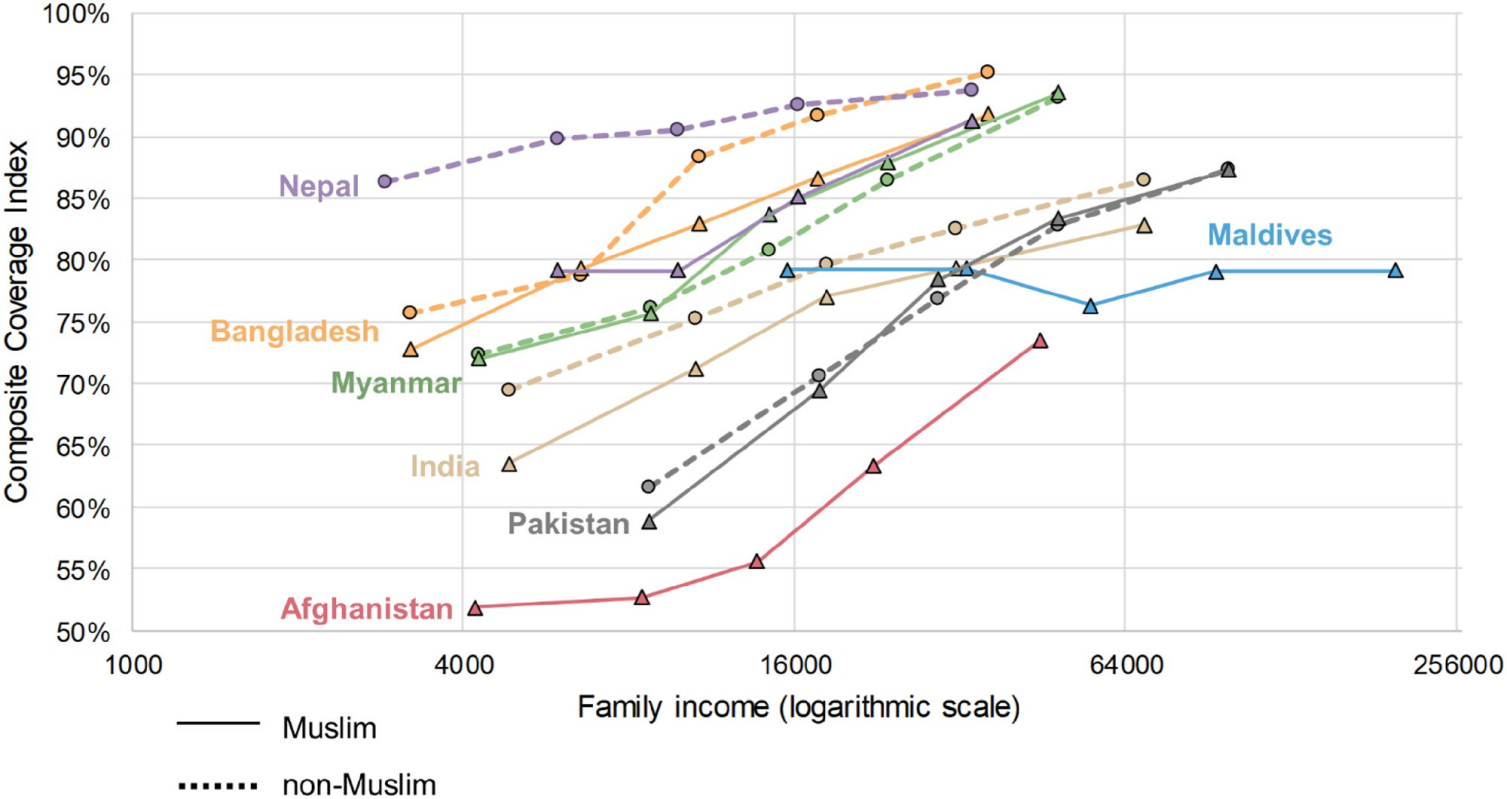
Relationship of religion and composite coverage index in 7 South Asian countries. Made with Natural Earth naturalearthdata.com.

In Fig 2, CCI is observed to be affected by family income, in addition to religion and country. Specifically, the higher the income, the higher the CCI rate for women, with the exception of the Maldives, which shows more equitable outcomes. Also of note is that, the lower the income, the larger the disparity between countries. This disparity becomes less the higher the income, regardless of country. Finally, the figure depicts that there are lower CCI rates among Muslim mothers than non-Muslim mothers in nearly every country and across every income bracket.

Finally, Table 4 summarizes the predictors of CCI among mothers of children aged 12–23 months, stratified by religion. Compared to Muslims, CCI was higher among Hindus (2.8%, 95% CI: 2.4%, 3.1%) and Buddhists or others (2.0%, 95% CI: 1.2%, 2.9%). CCI was comparable between Muslims and Christians. There was a U-shaped relationship between mother’s age and CCI–with lower CCI at younger ages and older ages, compared to mothers 20–24 years old. Mothers with decision-making autonomy had 18.8 percentage points higher CCI than those without (95% CI: 18.6%, 19.1%), and those believing beatings were not justified also had a higher CCI (1.7%, 95% CI: 1.3%, 2.0%). Mothers with higher levels of education or higher income had higher CCI.

**Table 4:** Predictors of composite coverage index (CCI) among mothers of children 12–23 months old in South Asia.

	Main effects model	Stratified model		
		Among Muslims	Among Non-Muslims	P-value*
**Child’s sex**				
** Female**	ref	ref	ref	
** Male**	0.2% (-0.1%, 0.4%)	0.6% (0.1%, 1.2%)	-0.1% (-0.5%, 0.2%)	0.0136
**Mother’s age**				
** 15–19**	-1.2% (-1.8%, -0.6%)	-2.3% (-3.4%, -1.3%)	0.1% (-0.7%, 0.9%)	0.0001
** 20–24**	ref	ref	ref	
** 25–29**	-1.5% (-1.8%, -1.2%)	-2.2% (-2.9%, -1.5%)	-1.2% (-1.6%, -0.8%)	0.0083
** 30–34**	-1.2% (-1.6%, -0.8%)	-1.4% (-2.2%, -0.6%)	-1.5% (-2.0%, -1.0%)	0.7875
** 35–39**	-2.7% (-3.3%, -2.1%)	-3.1% (-4.2%, -2.0%)	-2.4% (-3.2%, -1.6%)	0.3280
** 40–49**	-4.7% (-5.7%, -3.7%)	-4.2% (-6.0%, -2.4%)	-5.4% (-6.9%, -3.9%)	0.3200
**Mother’s religion**				
** Muslim**	ref	--	--	--
** Hindu**	2.8% (2.4%, 3.1%)	--	--	--
** Christian**	0.9% (-0.9%, 2.7%)	--	--	--
** Buddhist or other**	2.0% (1.2%, 2.9%)	--	--	--
**Mother’s education**				
** None**	-7.6% (-8.0%, -7.3%)	-11.0% (-11.8%, -10.3%)	-5.2% (-5.7%, -4.8%)	< .0001
** Primary**	-1.6% (-2.0%, -1.2%)	-2.8% (-3.6%, -2.0%)	-1.2% (-1.7%, -0.7%)	0.0003
** Secondary**	ref	ref	ref	
** Tertiary**	0.5% (0.0%, 0.9%)	1.3% (0.3%, 2.3%)	0.1% (-0.4%, 0.7%)	0.0242
**Decision-making autonomy**				
** No**	ref	ref	ref	
** Yes**	18.8% (18.6%, 19.1%)	16.4% (15.8%, 16.9%)	20.5% (20.2%, 20.8%)	< .0001
**Believes beatings justified**				
** Yes**	ref	ref	ref	
** No**	1.7% (1.3%, 2.0%)	2.0% (1.4%, 2.6%)	0.0% (-0.6%, 0.7%)	< .0001
**Log(family income)**	3.6% (3.5%, 3.8%)	4.5% (4.2%, 4.8%)	2.9% (2.7%, 3.1%)	< .0001

*Interaction term between Muslim and other variables

Table 4 shows a stratified model, where an interaction term between religion and other variables indicates whether there was a significant difference in the strength of association by a certain factor. In general, we found that the strength of association was greater for Muslims than non-Muslims. For example, compared to women with only a secondary education, Muslim women with no education had a CCI 11.0 percentage points lower, whereas this was only 5.2 percentage points lower for non-Muslim women (p <0.0001). Believing that beatings were justified was significantly related to CCI for Muslims (2.0%, 95% CI: 1.4%, 2.6%), but not for non-Muslims (0.0%, 95% CI: -0.6%, 0.7%, p <0.0001).

Specifically, for Muslim mothers, the mother’s education level, family income, decision-making autonomy about the use of contraception, and mothers who justified beatings are all statistically significant predictors of CCI value (p <0.0001). Mother’s age was less significant (p-values between 0.0001 and 0.7875) as was child’s sex (p = 0.0136).

## Discussion

Understanding how social practices such as religion affect health outcomes is an important first step to providing accessible solutions at the individual level to improve health outcomes at the group level. Based on the findings presented above, religion may play a role in maternal and child health outcomes. Given that Muslim and non-Muslim respondents had statistically significant differences in nearly every measure indicated by the CCI value, approaches that acknowledge cultural specifications will be necessary to further improve maternal and child health outcomes within the religiously diverse populations present in South Asian countries. This factor is doubly important given how family income did not change the results, indicating a heightened role for religion in dictating provision of antenatal and postnatal healthcare services, as depicted in Fig 2.

Similar to our findings, a recent multi country analysis conducted using DHS data in 15 Sub-Saharan African countries corroborates a correlation between religious affiliation and another health indicator—immunization coverage [29]. While that study does not directly measure maternal empowerment, it provides additional information relevant to our study—in that families who identified as Muslim had lower vaccination coverage for all their children compared to those who identified with other religions [29]. Similar to the results we obtained in this study, Costa et al. noted that non-Muslim families tended to be wealthier and have more education [29]. A similar multi country analysis in Sub-Saharan Africa by Carvajal-Aguirre et al. further illustrated the gap between coverage and content of both antenatal and postnatal care–factors which resulted in gaps in gaps in maternal and newborn health interventions [30]. Similar to the results presented here, Carvajal-Aguirre note the use of CCI indicators at a group level to analyze how women received antenatal and postnatal interventions, specifically noting that women with higher education and wealth status were more likely to receive these services [30].

The findings of this study build on existing work that illustrate the need to address social factors to improve health outcomes. To this end, Ahmed et al. demonstrated the importance of expanding maternal health services parallel to projects seeking to improve education, women’s empowerment, and reducing poverty [31]. Improving access to care and utilization of maternal health services will have positive downstream effects to improve children’s health outcomes, such as immunization rates, as well [27]. This work also confirms other DHS analyses, such as the study published by Ahmed et al. about stillbirths and neonatal mortality in Pakistan, which highlighted the importance of women’s empowerment in antenatal and postnatal care [32]. Our study illustrates a more robust understanding of the role of religion in pregnancy care and early childhood health outcomes, allowing for more generalizable results and the opportunity for further study in maternal health and measles vaccine interventions. Additionally, the knowledge that religion is a factor in these outcomes will allow for novel solutions to address these outcomes, while being sensitive to religious practices.

The results of this study can be further explained in a few ways. South Asian countries have a long history of colonization, violence, and other civil unrest that has had longstanding effects on population health [33]. Given this, the way in which pregnancy and early childhood healthcare is addressed has adapted, and could be shaped by religious practices. Specifically, for women who come from low income households, have less than primary school education, are married early, and lack empowerment in their reproductive decision making, their maternal and child health outcomes will be poorer than their counterparts. As Fig 1 highlights, areas with larger Muslim populations experience poorer health outcomes, and these individual factors may help explain why.

The role of religion is statistically significant and has implications beyond these South Asian countries. Understanding the role of religion in this contiguous region of the world may help explain outcomes in other countries with higher Muslim populations as well. It is important to consider the context of these results in the time that the DHS surveys were conducted in each of the countries–extreme natural disaster, armed conflict, and other humanitarian crises all were in play, which resulted in different policy actions across the different countries.

Given that much of this region of the world is unstable, there may be controversy over the fact that Muslim women have adverse outcomes compared to non-Muslim women in these countries. This is an important point to consider in order to better understand how the factors discussed in this work relate to CCI outcomes and how best to address them to facilitate better outcomes, while remaining understanding of current political conditions and societal norms [34].

### Strengths and limitations

There are many strengths to this study including the use of DHS data, which is in-depth, validated, and administered globally, increasing the generalizability of this study. Another strength of the study is the use of a validated outcome measure, the Composite Coverage Index, which provides a standardized measure to better understand maternal and child health.

This study does have limitations, the largest of which is the lack of religion data collected in the original DHS surveys for Afghanistan, Maldives, Myanmar, and Pakistan. Although we conducted a multiple imputation to complete some of this data, the assumptions used for the analysis may be under representative of actual religion preferences. Additionally, though the most recent DHS was utilized for every country, the years do vary, and the elapsed time could have an effect. Another limitation of the study is that maternal empowerment was defined based on the variables available across all countries’ datasets, and then only a few variables were included in the final analysis across the wide sample. We also acknowledge that other factors can contribute to maternal and child health outcomes, including ethnicity, and significant cultural norms that differ from region to region and country to country, which could impact the results presented here [35].

## Conclusions

In South Asia, maternal and child health outcomes continue to be a concern for many, but especially for those who are Muslim. Further study is needed to understand the role that religion plays, and what specifically is the interaction between religion, maternal empowerment, and maternal and child health outcomes. Additionally, future studies could be done to explore differences, if any, in Muslim majority countries between Sunni and Shia Muslims. Ultimately, understanding the relationship between religion and healthcare can lead to better interventions, and better outcomes, for maternal and child health in both the short and long term. Using the CCI as an outcome could be a simple solution to observe how health outcomes improve, and understanding the role that religion plays will allow for more accurate education and intervention.
